# Effect of cytokine-induced alterations in extracellular matrix composition on diabetic retinopathy-relevant endothelial cell behaviors

**DOI:** 10.1038/s41598-022-12683-7

**Published:** 2022-07-28

**Authors:** Meredith J. Giblin, Cayla D. Ontko, John S. Penn

**Affiliations:** 1 Department of Cell and Developmental Biology, Vanderbilt University, Nashville, USA; 2 Department of Molecular Physiology and Biophysics, Vanderbilt University, Nashville, USA; 3 Department of Ophthalmology and Visual Sciences, Vanderbilt University Medical Center, Nashville, USA

**Keywords:** Eye diseases, Mechanisms of disease, Inflammation, Extracellular matrix

## Abstract

Retinal vascular basement membrane (BM) thickening is an early structural abnormality of diabetic retinopathy (DR). Recent studies suggest that BM thickening contributes to the DR pathological cascade; however, much remains to be elucidated about the exact mechanisms by which BM thickening develops and subsequently drives other pathogenic events in DR. Therefore, we undertook a systematic analysis to understand how human retinal microvascular endothelial cells (hRMEC) and human retinal pericytes (hRP) change their expression of key extracellular matrix (ECM) constituents when treated with diabetes-relevant stimuli designed to model the three major insults of the diabetic environment: hyperglycemia, dyslipidemia, and inflammation. TNFα and IL-1β caused the most potent and consistent changes in ECM expression in both hRMEC and hRP. We also demonstrate that conditioned media from IL-1β-treated human Müller cells caused dose-dependent, significant increases in collagen IV and agrin expression in hRMEC. After narrowing our focus to inflammation-induced changes, we sought to understand how ECM deposited by hRMEC and hRP under inflammatory conditions affects the behavior of naïve hRMEC. Our data demonstrated that diabetes-relevant alterations in ECM composition alone cause both increased adhesion molecule expression by and increased peripheral blood mononuclear cell (PBMC) adhesion to naïve hRMEC. Taken together, these data demonstrate novel roles for inflammation and pericytes in driving BM pathology and suggest that inflammation-induced ECM alterations may advance other pathogenic behaviors in DR, including leukostasis.

## Introduction

Diabetic retinopathy (DR) is the leading cause of vision loss in working age Americans^1^. DR is categorized into two clinically distinct stages: the non-proliferative (NPDR) and proliferative (PDR)^2^. By the time a patient advances to PDR, the retina has sustained irreparable damage. Therefore, it is of great importance to understand the molecular mechanisms underlying the earliest stages of NPDR in hopes of preventing this damage from occurring. Numerous events occur on the molecular level that give rise to the characteristic lesions in NPDR. The roles of many of these events, including pericyte apoptosis, increased vascular permeability, leukostasis, and capillary dropout, in driving the pathogenic cascade have been extensively studied^2–5^. Conversely, although capillary basement membrane (BM) thickening has long been considered a characteristic event in NPDR^6^, relatively little is known about its contributions to DR pathogenesis^7^. Indeed, for a long time, BM thickening was believed to be a consequence, not a cause, of retinal pathology in DR. However, several pivotal studies have since demonstrated that BM thickening plays a role in driving other events in NPDR, including pericyte loss, acellular capillaries, and vascular hyperpermeability^8,9^. Additionally, a number of studies have expanded our understanding of how BM thickening progresses, demonstrating in in vitro and in vivo models that increased expression and deposition of key extracellular matrix (ECM) constituents, such as collagen IV, fibronectin, and laminin, are likely to advance the development of a thicker BM^7,10–23^. However, much remains to be understood about both the development of BM thickening and how BM thickening may contribute to the DR pathological cascade.

Among the limited studies of BM thickening in DR, the focus has been almost exclusively on the role of high glucose in stimulating changes in ECM expression and protein secretion by endothelial cells (EC). Although an important aspect of BM thickening, this approach ignores the potential contributions of other elements of the diabetic environment as well as other retinal cell types. First, while hyperglycemia is an important element, the diabetic environment is a complex mix of metabolic and inflammatory signals^5,24^. In recent years, the roles of dyslipidemia and chronic inflammation in DR pathogenesis have come under increased scrutiny^5,24–32^. However, few studies have explored the potential roles of lipid metabolites^33^ or inflammatory cytokines^17,34^ in promoting retinal BM thickening. Second, pericytes, Müller glia, and astrocytes are also key components of the neurovascular unit in addition to EC, and therefore could be involved in the dysregulation of BM deposition^35–39^. In particular, both pericytes and EC are considered important sources of microvascular BM constituents, and pericyte ECM deposition is known to be altered in other disease states^38,40–45^. The retina has the highest pericyte coverage of any vascular bed in the body, with pericytes present in a 1:1 ratio with EC, suggesting that pericytes could contribute significantly to retinal BM thickening.

Since cell–matrix interactions in the vascular unit represent complex mechanical and trophic signals, diabetes-induced changes in retinal BM could significantly alter the behavior of surrounding cells^7^. In other tissues, alterations in ECM constituency are known to contribute to impaired EC-pericyte communication, changes in microvascular permeability, inappropriate cell–matrix adhesions, and alterations in leukocyte extravasation^7,10,41,43,46–53^. Yet, despite this significant evidence from other tissues and disease states that cell–matrix interactions contribute to pathogenic cell behaviors, little is known about how changes in retinal BM might accelerate characteristic NPDR events. Increased leukocyte adhesion, or leukostasis, is a hallmark event of early NPDR, having been observed in both diabetic animals^25,54–56^ and patients^57–60^. It is well established that cell–matrix interactions and alterations in ECM constituency affect the leukocyte adhesion cascade^41,46,50,61^. Evidence has shown that both EC- and pericyte-derived ECM alterations can promote changes in expression of adhesion molecules and subsequent leukocyte behavior^41,52,53^. Overall, substantial evidence exists that changes in both EC- and pericyte-derived ECM can directly affect EC-leukocyte adherence, suggesting that diabetes-induced BM alterations might contribute to leukostasis in NPDR.

Therefore, in this study we sought to better understand how BM thickening might develop in response to the diabetic environment, and the potential of diabetes-related ECM changes to promote the increased leukocyte adherence characteristic of NPDR. First, we undertook a systematic analysis to determine how multiple retinal cell types change their expression patterns of ECM proteins under a variety of diabetes-relevant conditions. In this initial assessment, we chose to examine five ECM proteins: collagen IV, fibronectin, laminin β1, and the core proteins of the heparan sulfate proteoglycans agrin and perlecan. These proteins were selected based on their upregulation in diabetic animal models and human tissue and/or their central roles in BM function^8,9,11,12,16,19,20,62–66^. We utilized diabetes-relevant stimuli (DRS) which modeled the three major insults of the diabetic environment: hyperglycemia, dyslipidemia, and chronic retinal inflammation^2,5,24^. After surveying changes in the expression of key ECM constituents by human retinal endothelial cells (hRMEC) and human retinal pericytes (hRP) in response to DRS, we identified the inflammatory cytokines TNFα and IL-1β as having the most consistent and potent effects on ECM expression in retinal cells. Both TNFα and IL-1β are known to be elevated in the ocular fluids and retinal tissues of humans with DR and diabetic animals^5,67–76^. Furthermore, inhibition of TNFα or IL-1β signaling has been shown to attenuate retinal vascular pathologies in diabetic rodents^77–81^, and the nonsteroidal anti-inflammatory drug, Sulindac, prevented retinal capillary BM thickening in diabetic dogs^34^. We then utilized decellularized matrices derived from hRMEC or hRP under inflammatory conditions to investigate how changes in ECM might contribute to pathogenic behaviors in hRMEC. We demonstrated that inflammation-induced changes in ECM alone are sufficient to drive increased leukocyte adhesion molecule expression in, as well as increased leukocyte adherence to, naïve hRMEC.

## Methods

### hRMEC cell culture

Primary human retinal microvascular endothelial cells (hRMEC) were purchased from Cell Systems (Kirkland, WA); passages 3–8 were used for all experiments. Cultures were incubated at 37 °C, 5% CO_2_, and 20.9% O_2_ and 95% relative humidity. hRMEC were plated on attachment factor-(Cell Systems) coated 6-well plates. Cells were allowed to grow for 24–36 h in endothelial basal medium (EBM; Cell Systems) supplemented with 10% FBS (R&D Systems; Minneapolis, MN) and SingleQuots (Lonza, Inc.; Allendale, NJ) until a confluent monolayer was reached. The media was then switched to serum-reduced media [EBM supplemented with 2% FBS and 1 × antibiotic/antimycotic solution (Gibco; Carlsbad, CA)] for 48 h to allow sufficient time for cells to deposit ECM. In 2% FBS EBM, hRMEC were then treated with a variety of diabetes-relevant stimuli (DRS) for 48 h. Included were inflammatory cytokines (10 ng/mL TNFα, IL-1β, IL-6, IL-8, or 0.1% BSA in H_2_O vehicle), free fatty acids (250 μM palmitic acid, 100 μM oleic acid, 60 μM linoleic acid, or fatty acid-free BSA as vehicle), and high glucose conditions (5 mM or 25 mM d-glucose, with l-glucose used as an osmotic control). Free fatty acid concentrations were chosen for their physiological relevance. Total circulating levels of free fatty acids can be as high as 600 μM in diabetics^82–84^; more specifically, plasma palmitic acid levels were determined to be 234.9 ± 58.1 μmol/L in obese diabetic individuals fasted overnight^85^. Furthermore, these concentrations fall within ranges used in studies of retinal cell behaviors^86^ and in vitro studies of diabetes^87,88^. Likewise, our chosen cytokine concentrations fall within the range of concentrations tested in a variety of in vitro models of DR^17,89–92^. After treatment, cultures were collected for qRT-PCR analysis.

### hRP cell culture

Primary human retinal pericytes (hRP) were purchased from Cell Systems and maintained in normal glucose (5.5 mM) Dulbecco’s Modified Eagle Medium (DMEM; Gibco; Carlsbad, CA) supplemented with 10% FBS and SingleQuots on attachment factor-coated plates. Passages 6–8 were used for all experiments. At 75% confluency, hRP were treated with DRS at the same concentrations as hRMEC (see above) in serum-reduced media (2% FBS DMEM with 1 × antibiotic/antimycotic solution) for 24 h.

### Human Müller cell (hMC) isolation and culture

Primary human Müller cells (hMC) were isolated from human donor tissue (NDRI) within 24 h post-mortem, using an adapted protocol from previously developed methods^93^. Briefly, the retina was dissected from the eye cup and dissociated in DMEM supplemented with trypsin and collagenase. Following incubation in dissociation medium, hMC were cultured in DMEM supplemented with 10% FBS and 1 × antibiotic/antimycotic solution. Passages 4–6 were used for all experiments. At 70% confluency, hMC were treated in serum-reduced conditions (DMEM supplemented with 2% FBS and 1 × antibiotic/antimycotic solution) with DRS for 24 h at the same concentrations as described above for hRMEC.

### Conditioned media (CM) experiments

hMC were cultured in 6-well plates until 70% confluency, then cultured in serum-reduced conditions for 12 h before treatment. hMC were treated with TNFα (5 ng/mL or 10 ng/mL), IL-1β (1, 5, or 10 ng/mL), or corresponding vehicles for 2 h in serum-reduced media. Stimuli were removed and fresh 2% EBM media was placed onto hMC; CM was allowed to generate for 6 h before collection for treatment of hRMEC. Once cultures reached confluence, hRMEC media was changed to 5% FBS EBM (plus SingleQuots) for 24 h prior to treatment with hMC CM. hRMEC were treated with hMC CM for 48 h prior to collection for qRT-PCR analysis.

### mRNA expression

Total RNA was isolated from cells using an RNeasy mini kit (Qiagen; Valencia, CA). cDNA was reverse transcribed using the High-Capacity cDNA Archive Kit (Applied Biosystems; Carlsbad, CA) according to the manufacturer’s instructions. Quantitative RT-PCR was performed by co-amplification of the genes of interest [human *COL4A1*, *FN*, *LAMB1, AGRN,* or *HSPG2* (perlecan)] vs. TATA-binding protein (TBP; endogenous normalization control), using gene-specific TaqMan Gene Expression Assays (Table 1) according to the manufacturer’s instructions (Applied Biosystems). Full sequence and other primer information can be found on the manufacturer’s website. Expression data were analyzed by the comparative Ct method. Experiments were performed using a minimum of 3 biologically independent samples as well as technical replicates for each sample.

**Table 1:** Gene expression assays used in expression studies.

Target	Primer	Species
*TBP*	Hs00427620_m1	Human
*COL4A1*	Hs00266237_m1	Human
*FN1*	Hs00365052_m1	Human
*LAMB1*	Hs01055967_m1	Human
*AGRN*	Hs00394748_m1	Human
*HSPG2*	Hs01078536_m1	Human
*VCAM1*	Hs1003372_m1	Human
*ICAM1*	Hs00164932_m1	Human
*SELE*	Hs00174057_m1	Human

Gene-specific TaqMan Gene Expression Assays were obtained from Applied Biosystems (Carlsbad, CA).

### Decellularization experiments

For experiments with hRMEC-derived matrices, media was changed to EBM supplemented with 5% FBS and SingleQuots for 24 h once cultures reached 100% confluency. hRMEC were then treated for 48 h with 10 ng/mL TNFα or IL-1β in 5% EBM. For experiments with hRP-derived matrices, hRP were treated at 75% confluency with 10 ng/mL TNFα or IL-1β for 48 h in serum-reduced media. Subsequently, hRMEC or hRP cultures were decellularized using methods adapted from published protocols^94–96^. Briefly, cultures were washed once with PBS before treatment with decellularization buffer [20 mM NH_4_OH and 0.5% Triton-X (vol/vol) in PBS (with Ca and Mg)] at 37 °C; cultures were monitored visually for complete decellularization (5–10 min). Decellularized ECM was washed three times by adding PBS in equal volume to the dish to dilute cellular debris and then removing half of the volume^96^. Naïve hRMEC were plated to settle at a density of 85% confluency onto the hRMEC- or hRP-derived matrices and allowed to settle onto the decellularized ECM for 16 h before collection for qRT-PCR analysis. qRT-PCR analysis was carried out as described above, looking at the expression of *SELE* (E-selectin)*, ICAM1,* and *VCAM1.*

### Static adhesion assay (SAA)

hRMEC or hRP were plated onto attachment factor-coated 24-well plates and allowed to settle at room temperature for 15 min (to ensure proper monolayer formation). Cells were treated as indicated above for decellularization experiments. After treatment, cultures were decellularized and decellularized ECM was washed three times as above. Naïve hRMEC were plated to settle as a confluent monolayer and allowed to settle for 8 h before SAA was performed. The SAA protocol was adapted from published protocols^52,53^; briefly, human peripheral blood mononuclear cells (PBMC; Precision for Medicine; Fredrick, MD) were stained with NucBlue (Thermo Fisher; Waltham, MA) for 20 min at 37 °C at the manufacturer’s suggested concentration and then spun down and resuspended. PBMC were added to each well at a concentration of 125,000 cells/cm^2^ and allowed to settle onto monolayers for 30 min at 37 °C. Subsequently, PBMC were removed and cultures were gently washed three times with warm PBS to remove non-adherent cells. Cultures were then fixed in 1% PFA (Electron Microscopy Sciences; Hatfield, PA) for 10 min at 37 °C. Cultures were washed once with warm PBS. Three fields were randomly selected per well and adherent PBMC were counted. Each data point represents the average number of adherent leukocytes for the three captured fields per well and is reported as adherent cells per mm^2^.

### Statistical analysis

All data were analyzed with Prism software (GraphPad; La Jolla, CA). Student’s *t*-test and analysis of variance (ANOVA) with Tukey’s multiple comparisons post hoc analysis was used; values of p < 0.05 were considered statistically significant. The ROUT method was utilized to identify outliers.

## Results

### Inflammatory cytokines TNFα and IL-1β cause significant changes in the expression of key ECM constituents in hRMEC

The limited focus of previous studies on high glucose driving BM thickening ignores the potential contributions that chronic inflammation or diabetic dyslipidemia may also make. Therefore, we utilized a number of DRS to model different elements of the diabetic environment, specifically: TNFα, IL-1β, IL-6, and IL-8 to model chronic retinal inflammation^5,67–76,97–101^; palmitic, oleic, and linoleic acid to model diabetic dyslipidemia^24,82–85,102,103^; and high glucose to model diabetic hyperglycemia^104^. Using these DRS, we undertook a systematic assessment to determine how different elements of the diabetic environment alter hRMEC expression of key ECM constituents: collagen IV, fibronectin, laminin β1, and the core proteins of the heparan sulfate proteoglycans agrin and perlecan. Under our experimental conditions, we did not observe any significant changes in the expression of four of the five ECM proteins under high glucose conditions (Table 2); high glucose only caused a modest significant increase in perlecan expression (1.20-fold, p = 0.0203). All three free fatty acids caused significant decreases in fibronectin, while linoleic acid caused a small but significant increase in perlecan expression. No other significant changes were observed with free fatty acid treatment. Interestingly, we found that the cytokines TNFα and IL-1β were most consistent in causing alterations in ECM expression across multiple proteins. TNFα caused 2.13- (p = 0.0002), 0.68- (p = 0.0032), 2.31- (p < 0.0001), and 0.56-fold (p = 0.0075) changes in the expression of collagen IV, fibronectin, agrin, and perlecan, respectively. Similarly, IL-1β caused 2.03- (p < 0.0001) and 1.44-fold (p = 0.0036) changes in the expression of collagen IV and agrin, respectively.

**Table 2:**
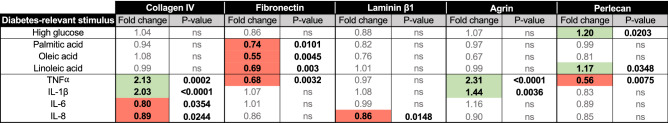
Diabetes-relevant stimuli cause significant effects in ECM expression by hRMEC.

All statistical comparisons used t-tests between the DRS-treated group and its respective vehicle, except for the case where normal glucose, l-glucose (osmotic control), and high glucose were compared. In that case ANOVA with Tukey post-hoc was used. For simplicity, only l-glucose vs high glucose is reported here. Expression data are reported as fold induction over vehicle. Red cells highlight statistically significant decreases in expression; green cells highlight statistically significant increases in expression.

### Inflammatory cytokines are also the most potent inducers of changes in ECM expression in hRP

Pericytes are known contributors to both normal and pathogenic deposition of vascular BM, and yet their role in BM thickening in DR has been previously ignored^40–45^. Therefore, we also completed a systematic assessment of how hRP alter expression of key ECM proteins under DRS treatment. Interestingly, similar trends were observed in the types of DRS that caused significant alterations in expression of ECM constituents by hRP as were seen in hRMEC. Specifically, high glucose, IL-6, and IL-8 did not cause any significant changes, while TNFα and IL-1β caused potent alterations in hRP expression of most ECM constituents (Table 3). TNFα caused 2.76- (p < 0.0001), 0.65- (p = 0.0027), 3.27- (p < 0.0001), and 1.32-fold (p = 0.0012) changes in the expression of collagen IV, laminin β1, agrin, and perlecan, respectively. Similarly, IL-1β caused alterations in the expression of all five constituents under study, specifically: 1.83- (p = 0.002), 0.81- (p = 0.0311), 0.59- (p = 0.0025), 1.18- (p = 0.0166), and 1.18-fold (p = 0.0099) changes in the expression of collagen IV, fibronectin, laminin β1, agrin, and perlecan, respectively. Interestingly, palmitic acid caused significant decreases in the expression of all five constituents. Oleic acid caused significant decreases in fibronectin, laminin β1, and perlecan, while linoleic acid only caused a significant decrease in laminin β1 expression. We noted many interesting findings in this survey, which will provide the basis for future studies. However, based on the increasing importance of inflammation in DR and the lack of investigations into its role in BM thickening, we chose to narrow our focus to TNFα and IL-1β in subsequent experiments.

**Table 3:**
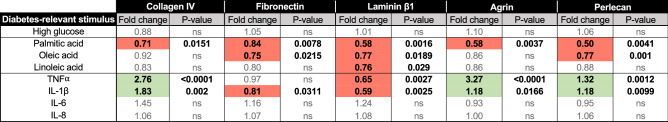
Diabetes-relevant stimuli cause significant effects in ECM expression by hRP.

All statistical comparisons used t-tests between the DRS-treated group and its respective vehicle, except for the case where normal glucose, l-glucose (osmotic control), and high glucose were compared. In that case ANOVA with Tukey post-hoc was used. For simplicity, only l-glucose vs high glucose is reported here. Expression data are reported as fold induction over vehicle. Red cells highlight statistically significant decreases in expression; green cells highlight statistically significant increases in expression.

### hMC cause only minor alterations in ECM expression but hMC-derived inflammation can influence hRMEC ECM expression

**Figure 1:**
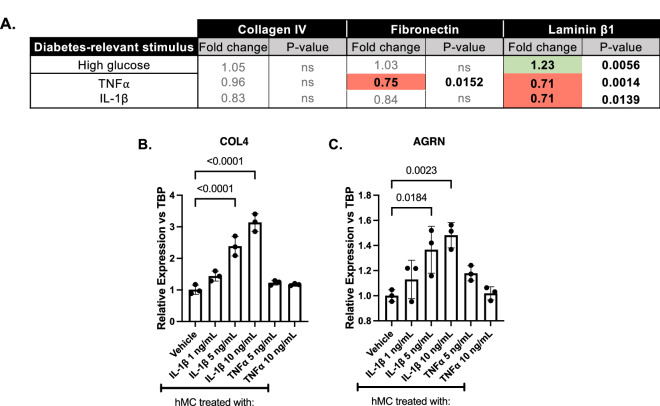
hMC-derived inflammation can influence hRMEC ECM expression. (**A**) All statistics are t-tests between the DRS-treated group and its respective vehicle. Expression data are reported as fold induction over vehicle. Red cells highlight statistically significant decreases in expression; green cells highlight statistically significant increases in expression. (**B**,**C**) Conditioned media (CM) was collected from IL-1β- or TNFα-treated hMC. hRMEC were treated with CM from hMC and then collected and assayed for expression of collagen IV or agrin. Expression data are reported as fold induction over vehicle with bars representing mean ± SD (n = 3 for all groups).

We performed a small pilot study utilizing key DRS (high glucose, TNFα, and IL-1β) to investigate if hMC similarly altered their expression of ECM constituents under DRS. No significant changes in collagen IV expression were observed and only TNFα caused a significant decrease in fibronectin expression (Fig. 1A). All three DRS caused changes in laminin β1 expression with high glucose causing a slight increase (1.23-fold, p = 0.0056) and both TNFα and IL-1β causing a 0.71-fold decrease (p = 0.0014 and p = 0.0139, respectively) (Fig. 1A). Overall, the changes observed were smaller compared to changes observed in hRMEC and hRP and thus we did not pursue hMC in subsequent decellularization experiments. However, evidence suggests that Müller cells are a primary driver of retinal inflammation^29,32,105–109^. Conditioned media (CM) from TNFα-treated hMC caused no significant changes in collagen IV or agrin expression in hRMEC (Fig. 1B,C). Conversely, when hRMEC were treated with CM from IL-1β-treated hMC, expression of both collagen IV and agrin increased in a dose-dependent manner. At the highest dose, CM from IL-1β-treated hMC caused a 3.14- (p < 0.0001) and 1.48-fold (p = 0.0023) increase in collagen IV and agrin expression, respectively (Fig. 1B,C).

### ECM deposited by cytokine-treated hRMEC causes induction of adhesion molecules in naïve hRMEC

**Figure 2:**
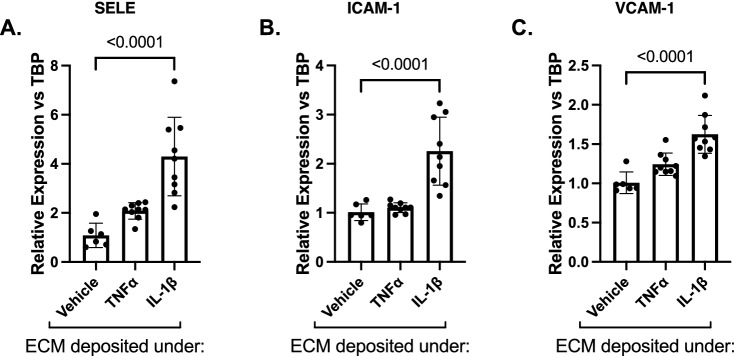
ECM deposited by cytokine-treated hRMEC causes induction of adhesion molecules in naïve hRMEC. Naïve hRMEC were plated onto ECM derived from TNFα- or IL-1β-treated hRMEC. Cells were collected and assayed for expression of (**A**) E-selectin (gene name: SELE), (**B**) ICAM-1, or (**C**) VCAM-1. Expression data are reported as fold induction over vehicle with bars representing mean ± SD (vehicle: n = 6; TNFα: n = 9; IL-1β: n = 9).

Numerous studies have demonstrated that changes in the constituency and/or biomechanics of vascular BM drive increased leukocyte adhesion to vascular endothelium^41,46,49,50,52,53,61^. Therefore, based on our finding that DR-relevant cytokines significantly alter the expression of ECM proteins in retinal cells, we hypothesized that this inflammation-conditioned ECM contributes to the development of retinal leukostasis. To investigate how inflammation-induced changes in ECM could alter the behavior of naïve hRMEC, we adopted previously described decellularization techniques. In these experiments, hRMEC were first treated with cytokines TNFα or IL-1β. Cultures were then decellularized, and naïve hRMEC were plated on the vehicle- or cytokine-conditioned ECM. Naïve hRMEC were subsequently collected to analyze expression of key leukocyte adhesion proteins, E-selectin (gene name *SELE)*, ICAM-1, and VCAM-1. The naïve hRMEC received no treatment; the only difference between the control and experimental cultures was the conditioned ECM upon which they attached. When naïve hRMEC were grown on ECM derived from TNFα-treated hRMEC, 2.08- and 1.24-fold increases in E-selectin and VCAM-1 expression were observed, respectively; however, results did not reach statistical significance. When naïve hRMEC were plated on ECM derived from IL-1β-treated hRMEC, 4.30- (p < 0.0001), 2.26- (p < 0.0001), and 1.62-fold (p < 0.0001) increases in E-selectin, ICAM-1, and VCAM-1 expression were observed, respectively, relative to hRMEC plated on ECM derived from vehicle-treated hRMEC (Fig. 2A–C).

### Cytokine-conditioned ECM derived from hRMEC is sufficient to drive increased PBMC adhesion to naïve hRMEC

**Figure 3:**
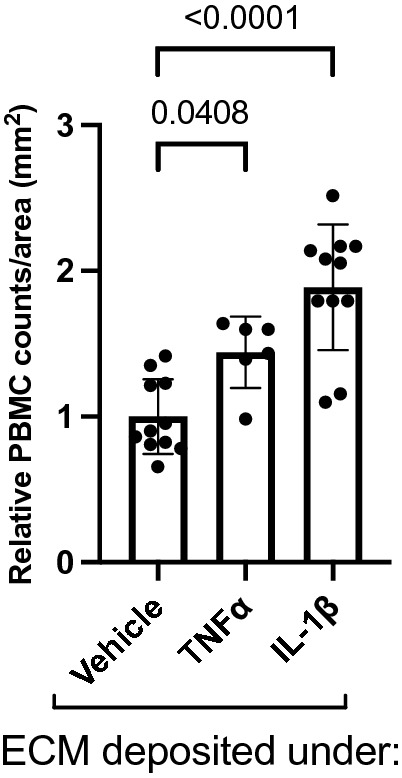
ECM derived from TNFα- or IL-1β-treated hRMEC causes increased PBMC adhesion. Naïve hRMEC monolayers were plated onto ECM derived from TNFα- or IL-1β-treated hRMEC. PBMC were added to naïve hRMEC, allowed to adhere, and washed to remove non-adherent cells. Adherent PBMC were counted in 3 regions per well and averaged. Average counts were then divided by count area. Data are reported as fold induction over vehicle with bars representing mean ± SD (vehicle: n = 11; TNFα: n = 6; IL-1β: n = 11).

We next sought to determine if the changes seen in adhesion molecule expression resulted in corresponding changes in leukocyte adhesion behavior. To answer this question, we utilized static adhesion assays to test PBMC adhesion to monolayers grown on cytokine-conditioned ECM. When hRMEC monolayers were grown on TNFα-conditioned ECM, there was a 1.44-fold (p = 0.0408) increase in PBMC adhesion to the naïve hRMEC. Likewise, when monolayers were grown on IL-1β-conditioned ECM, there was a 1.89-fold (p < 0.0001) increase in PBMC adhesion (Fig. 3).

### ECM derived from cytokine-treated hRP causes induction of hRMEC adhesion molecules

**Figure 4:**
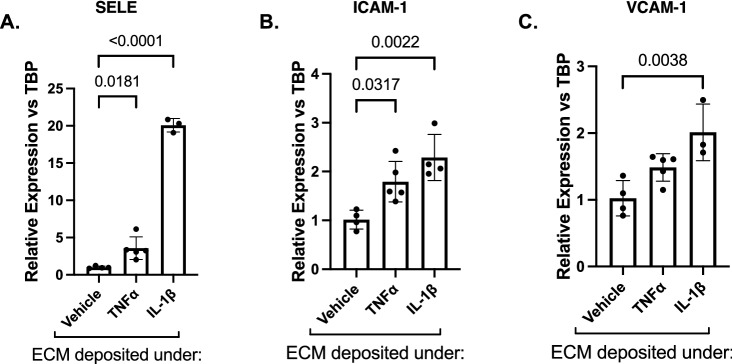
ECM derived from cytokine-treated hRP causes induction of hRMEC adhesion molecules. Naïve hRMEC were plated onto ECM derived from TNFα- or IL-1β-treated hRP. hRMEC were collected and assayed for expression of (**A**) E-selectin (gene name: SELE), (**B**) ICAM-1, or (**C**) VCAM-1. Expression data are reported as fold induction over vehicle with bars representing mean ± SD (vehicle: n = 4; TNFα: n = 5; IL-1β: n = 4).

Based on our findings that hRP, similar to hRMEC, alter expression of ECM constituents under TNFα or IL-1β treatment, we investigated whether hRP-derived ECM could also alter EC-leukocyte behaviors. Naïve hRMEC were plated onto TNFα- or IL-1β-conditioned ECM deposited by hRP, and expression of E-selectin, ICAM-1, and VCAM-1 was measured. When naïve hRMEC were plated on ECM derived from TNFα-treated hRP, 3.57- (p = 0.0181) and 1.79-fold (p = 0.0317) increases in E-selectin and ICAM-1 expression were observed, respectively (Fig. 4A–C). Likewise, when naïve hRMEC were plated on ECM derived from IL-1β-treated hRP, 20.08- (p < 0.0001), 2.29- (p = 0.0022), and 2.01-fold (p = 0.0038) increases in E-selectin, ICAM-1, and VCAM-1 expression were observed, respectively.

### Cytokine-conditioned matrix derived from hRP is sufficient to drive increased PBMC adhesion to naïve hRMEC

**Figure 5:**
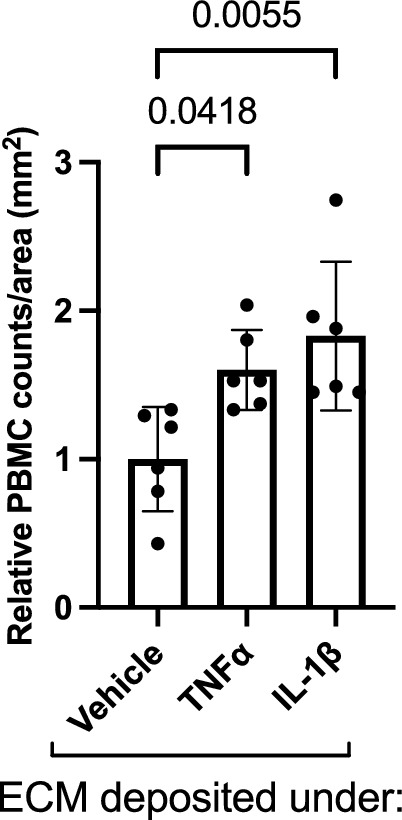
ECM derived from TNFα- or IL-1β-treated hRP causes increased PBMC adhesion. Naïve hRMEC monolayers were plated onto ECM derived from TNFα- or IL-1β-treated hRP. PBMC were added to naïve hRMEC, allowed to adhere, and washed to remove non-adherent cells. Adherent PBMC were counted in 3 regions per well and averaged. Average counts were then divided by count area. Data are reported as fold induction over vehicle with bars representing mean ± SD (vehicle: n = 6; TNFα: n = 6; IL-1β: n = 6).

Finally, we sought to again investigate if the changes seen in adhesion molecule expression resulted in corresponding changes in leukocyte adhesion behavior utilizing static adhesion assays. When hRMEC monolayers were grown on TNFα-conditioned ECM derived from hRP, there was a 1.60-fold (p = 0.0418) increase in PBMC adhesion to the naïve hRMEC (Fig. 5). Likewise, when monolayers were grown on IL-1β-conditioned ECM derived from hRP, there was a 1.83-fold (p = 0.0055) increase in PBMC adhesion.

## Discussion

In this report, we offer several new contributions to our understanding of BM thickening in DR. First, we demonstrate that conditions designed to mimic chronic inflammation (direct stimulation with inflammatory cytokines) caused larger alterations in expression of key ECM constituents by both hRMEC and hRP than did conditions that mimic hyperglycemia and dyslipidemia. Second, we demonstrate that the alterations in ECM that occur under these inflammatory conditions were alone sufficient to drive pathogenic behaviors in naïve EC. Third, we demonstrate that, similar to EC, pericytes both altered their expression of ECM constituents under inflammatory conditions and also deposited conditioned ECM that caused increased leukocyte adhesion in naïve EC, suggesting that pericytes may play a significant role in BM thickening.

To our knowledge, ours is the first systematic survey defining how different DRS affect retinal cell expression of ECM constituents. Our qRT-PCR results demonstrate that, in both hRMEC and hRP, cytokines TNFα and IL-1β caused the largest changes in expression of key ECM constituents. The DRS designed to model diabetic hyperglycemia and dyslipidemia were either ineffective or far less effective in eliciting significant alterations than TNFα or IL-1β. These findings are consistent with studies from our lab and others suggesting that chronic inflammation, particularly manifested via the cytokines TNFα and IL-1β, is a primary force behind the early pathogenic events in DR^28,30–32,77–81,89^. Therefore, in subsequent experiments, we focused our efforts on the role of inflammatory cytokines in driving ECM alterations. However, it is worth noting that the free fatty acids caused significant decreases in expression of fibronectin in hRMEC and fibronectin, laminin β1,  and perlecan in hRP. In particular, palmitic acid caused significant decreases in the expression of all five ECM proteins under study in hRP. While these results were surprising, published evidence has demonstrated that free fatty acids can suppress ECM expression in both in vitro and in vivo models^110,111^. When this free fatty acid-induced suppression of ECM expression is considered in combination with the increased expression observed under cytokine treatment, and the fact that, in vivo*,* the diabetic environment encompasses a complex mix of inflammatory and metabolic signals, these results suggest significant and, at times, contrasting alterations in the deposition of ECM. Changes in the ratios of these ECM constituents could represent major shifts in overall constituency, affecting stiffness of the vascular BM, thereby significantly contributing to altered cell–matrix interactions in DR. Although we chose to focus on cytokine-induced changes in the remainder of this study, future studies will focus on the potential contributions of these dyslipidemia-induced changes in ECM to DR pathology. Finally, we are aware that the failure of high glucose to stimulate altered expression of most ECM constituents in our experiments contradicts previously published studies^13–15,18,21–23^. However, previous studies had a number of limitations, including lack of an osmotic control for d-glucose treatment, ignoring the significant osmotic effects of high glucose, or use of rodent or non-retinal EC, despite hRMEC being known to be phenotypically unique from other common EC lines^112–115^. In our studies, we were careful to utilize osmotic controls and primary human retinal microvascular cells. Furthermore, previous studies often treated EC with high glucose for long exposures (days or weeks); we chose relatively short exposure times (48 h) to investigate more immediate expression changes in response to these stimuli. Although we did not find high glucose to cause notable changes in ECM expression during this time window, we do not mean to suggest that hyperglycemia plays no role in promoting retinal BM thickening. Indeed, the diabetic environment offers a complex mixture of stimuli, with no single stimulus acting alone at one time. Therefore, we tested whether high glucose pre-treatment might potentiate the IL-1β-induced ECM expression observed here. When we pre-treated hRMEC with high glucose, IL-1β-induced ECM expression was not potentiated in hRMEC (data not shown). These results are consistent with our data in Table 2, showing that inflammatory stimuli, but not high glucose, elicit changes in the expression of ECM constituents. Nevertheless, we have previously demonstrated that human Müller cells exhibit higher inflammatory responsivity to palmitic acid treatment when pre-treated with high glucose, suggesting an interplay between multiple DRS^27^. High glucose could play a similar role in promoting BM thickening and future studies will investigate combinations of high glucose and free fatty acids.

Existing data have focused almost exclusively on the role of EC in the development of BM thickening, ignoring the potential contributions of other retinal cell types in the neurovascular unit. Since pericytes are highly abundant in the retina and contribute to both normal and pathogenic deposition of BM in other systems^40–45^, we hypothesized that pericytes might also be critical contributors to BM thickening in DR. Only one other study has specifically addressed the potential of pericytes to contribute to retinal BM thickening, and it found that pericyte-derived matrices had ten-fold higher levels of fibronectin than EC-derived matrices^23^. Furthermore, a recent report utilizing RNA sequencing to study pericytes isolated from diabetic mice identified “Enhancers of ECM synthesis” as one of the top hits in a gene set enrichment analysis^116^. This study was the first to provide a detailed analysis of the pericyte transcriptome in diabetic animals and further supports our hypothesis that pericytes play a major role in retinal BM thickening. Our results demonstrate that both TNFα and IL-1β caused increased expression of collagen IV, agrin, and perlecan and decreased expression of laminin β1 in hRP. Interestingly, TNFα-treated hRP demonstrated higher relative fold changes of both collagen IV and agrin expression than hRMEC. These data are the first evidence that retinal pericyte expression of ECM constituents is altered under DRS and suggest that pericytes contribute to the development of BM thickening. In addition to pericytes, we considered the potential contributions of hMC since Müller cells are also in direct contact with the vascular BM and limited evidence points to their role in depositing ECM^35^. We performed a brief survey with hMC utilizing a limited set of DRS. Although we did note significant changes in the expression of laminin β1, we chose not to pursue hMC further in this study since, overall, the changes were minor compared to those seen in hRMEC and hRP. However, we and others have evidence that Müller cells act as a primary driver of chronic retinal inflammation and serve as the major source of retinal TNFα and IL-1β in diabetes^29,32,89,105–109^. Interestingly, when hRMEC were treated with conditioned media (CM) from hMC treated with IL-1β, significant increases in collagen IV and agrin expression were observed in a clear dose-dependent manner. We also tested whether CM from hMC treated with TNFα or palmitic acid could similarly drive increased ECM expression in hRMEC, however increased ECM expression was not observed in hRMEC treated with hMC CM from either stimuli (Figure 1; data not shown). Still, the IL-1β CM results suggest that although Müller cells may not directly contribute to BM thickening, Müller cell-dependent inflammation almost certainly contributes to the initiation and progression of retinal BM thickening in surrounding cells. Future studies will include more detailed investigations into this cellular interplay.

Despite an increasing focus on inflammation in DR progression^5,25,26^, few studies have assessed the effects of inflammation on retinal matrix deposition. Our systematic analysis of DRS raises many questions but here we chose to focus on the implications of our results with cytokine stimulation. First, in our studies, both hRMEC and hRP demonstrated significantly increased expression of collagen IV in response to treatment with TNFα and IL-1β, results directly in line with multiple studies that have demonstrated increased collagen IV synthesis in in vitro and in vivo models of DR^7,10–17^. Second, the decreased perlecan expression seen in TNFα-treated hRMEC is in line with the results of a mass spectrometry study comparing the constituencies of retinal vascular BM isolated from non-diabetic and diabetic donors, wherein perlecan was identified as a constituent that was more abundant in the non-diabetic BM than the diabetic BM^64^. We noted significant changes in agrin and perlecan expression under TNFα and IL-1β in both hRMEC and hRP. These findings have important implications for retinal cell–matrix interactions; indeed, we specifically chose to include agrin and perlecan in this study because heparan sulfate proteoglycans play such key roles in the structure and function of BM. Heparan sulfate proteoglycans provide important structural contributions to BM integrity, serve as depots of regulatory factors such as cytokines and growth factors, and facilitate the establishment of chemokine gradients for leukocyte recruitment and homing in tissues^65,66^, yet their role in retinal BM thickening has been largely overlooked. Finally, our findings that fibronectin expression either decreased (hRMEC) or remained unchanged (hRP) under DRS were surprising as multiple studies have identified increases in fibronectin in diabetic rodents and humans^12,19,20,62–64^. However, our studies only capture expression changes at a single time point, highlighting an important limitation of qRT-PCR studies. For this reason, we have completed studies utilizing quantitative mass spectrometry to investigate changes in ECM deposition under both TNFα and IL-1β treatment (manuscript in preparation). It is interesting to note that for both hRMEC and hRP, TNFα caused more potent changes in ECM expression than IL-1β. However, in the decellularization experiments, IL-1β-conditioned ECM caused more potent changes in adhesion molecule expression and leukocyte adhesion. These results suggest that important ECM properties are governed by more than our five key ECM constituents, and further necessitates a quantitative mass spectrometry approach.

Although vascular BM thickening has long been considered a characteristic event in early DR^6^, it has been overlooked for its potential to advance other events in the pathologic cascade. However, a series of in vivo studies in rodent models of diabetes demonstrated that antisense oligonucleotides targeting key BM constituents not only reduced capillary BM thickening but also reduced other early NPDR events, including pericyte loss, acellular capillaries, and vascular hyperpermeability^8,9^. Additionally, in vitro studies have demonstrated that high glucose-induced changes in ECM can alter EC permeability and apoptosis as well as pericyte apoptosis^9,15,117,118^. These studies provided clear evidence that BM thickening is an active participant in the DR pathologic cascade and invited further exploration into the ways in which BM alterations could elicit pathogenic vascular cell behavior. Leukostasis is an important and well-studied event in NPDR and is a consequence of increased endothelial expression of the leukocyte adhesion proteins E-selectin, ICAM-1, and VCAM-1. Adherent leukocytes can occlude capillaries leading to downstream ischemia or amplify local inflammation and release of pro-apoptotic factors^4,5,25,54,55,57^. Numerous studies have demonstrated that cell–matrix interactions can produce changes in leukocyte adhesion^41,46,50,61^, including two studies in retinal EC demonstrating that changes in ECM stiffness associated with high glucose treatment were sufficient to increase monocyte adhesion to EC^52,53^. Here, we demonstrated that when naïve hRMEC are grown on ECM deposited by IL-1β-treated hRMEC, they increase expression of leukocyte adhesion molecules, and this increased expression is sufficient to cause increased PBMC adhesion. Our results from TNFα-conditioned ECM showed increased trends in adhesion molecule expression that did not reach significance. Yet, increased PBMC adhesion was observed, suggesting that TNFα can also drive ECM alterations sufficient to promote pathogenic cell behavior. We are the first to examine chronic retinal inflammation as a contributor to altered cell–matrix dynamics in retinal cells. Coupled with what is known about the effects of glucose on cell–matrix interactions^52,53^, these data suggest that the diabetic environment promotes significant alterations in cell–matrix dynamics that subsequently contribute to pathogenic cell behaviors.

Like EC-derived ECM, pericyte-derived ECM is known to alter cell behavior in a number of other systems^40–45^. Of particular relevance, alterations in ECM deposition by placental microvascular pericytes under pro-inflammatory conditions caused increased EC expression of ICAM-1 and neutrophil transmigration^41^. However, the potential role of pericyte-derived ECM in pathogenic retinal cell behavior has not previously been considered. When naïve hRMEC were grown on ECM deposited by TNFα- or IL-1β-treated hRP, significant increases in E-selectin, ICAM-1, and VCAM-1 expression were observed. Furthermore, naïve hRMEC monolayers showed increased PBMC adherence when grown on the TNFα- or IL-1β-conditioned hRP-derived ECM. These data demonstrate that, similar to EC, inflammation-induced changes in ECM derived from hRP alone are sufficient to elicit pathogenic leukocyte adhesion behavior. These studies are the first to demonstrate that hRP-derived matrices cause alterations in hRMEC behavior relevant to DR. These results are particularly exciting since there is increasing focus on the role of pericytes in vascular inflammation and leukocyte extravasation^119^. Pericytes release leukocyte chemoattractants, express adhesion molecules, and control the direction of leukocyte motility through the vascular BM^38,43,119–122^. Therefore, it is of interest to explore how diabetes-relevant changes in ECM alter the path, timing, and success of leukocyte extravasation along pericytes processes and through the vascular BM. Understanding these complicated dynamics between pericytes, EC, vascular BM, and leukocytes will be key to fully elucidating the molecular mechanisms of early DR.

It has long been known about non-ocular tissues that chronic inflammation leads to significant alterations in the constituency and functionality of ECM^46^. Therefore, it was not surprising that we found inflammatory cytokines to cause the largest and most consistent changes in ECM expression and that cytokine-conditioned ECM elicits associated changes in EC behavior. Future studies will endeavor to identify the molecular, biochemical, or biophysical property shifts in ECM deposited under inflammatory conditions that underlie these shifts in EC/leukocyte adhesion behavior. There are a number of possible contributors. First, previous studies have demonstrated that shifts in the constituency of ECM, particularly in the ratios of key proteins, can cause shifts in the behavior of neighboring cells^41,50,123^. In a number of cases, we observed contrasting changes in expression between different ECM constituents. For instance, TNFα caused increased agrin, but decreased perlecan, expression in hRMEC. Contrasting expression changes can amplify differences in the ratios of constituents in diabetic retinal BM, fundamentally altering its architecture and therefore its functionality^123,124^. Second, alterations in the ratios and constituency of BM constituents can cause significant shifts in ECM stiffness. Changes in BM stiffness profoundly alter the behavior of adjacent cells sensing those stiffness shifts, in turn accelerating disease pathology^50,124,125^. Of particular relevance, previous studies have demonstrated that changes in ECM stiffness cause similar increases in leukocyte adherence^52,53^. Therefore, it would be of value to measure stiffness alterations in our cytokine-conditioned ECM. Finally, the ECM can serve as a reservoir of secreted and trapped cytokines and growth factors, so it is possible that these matrix-associated proteins contribute to the observed changes. Although in our in vitro studies we did not identify any increased inflammatory mediators in the ECM, it remains a possibility in the more complex in vivo retinal BM. Consequently, a more global and detailed understanding of how ECM deposition is altered under inflammatory conditions will be necessary to elucidate the exact mechanisms of action underlying the changes we have observed in naïve EC.

Our findings point to a number of areas for future investigation which were outside the scope of this study. Included are: how inflammation-induced changes in ECM alter matrix stiffness, how dyslipidemia contributes to BM thickening, and the molecular mechanisms underlying adhesion protein induction in naïve hRMEC. Additionally, future studies will interrogate whether inflammation-induced changes in ECM could be involved in other NPDR events, and whether similar mechanisms of action underlie these other events. It is important to acknowledge the challenge of modeling a chronic disease that develops over decades using in vitro methods that take only hours or days, and the limitations inherent in the extrapolation of these methods to disease events. However, BM is a particularly difficult structure to study in vivo, particularly when multiple cells contribute to its construction and maintenance, and we believe that our in vitro environment offers a reasonable first step to identifying important cell–matrix relationships. We also recognize that the changes in expression of ECM constituents, expression of adhesion molecules, and leukocyte adhesion observed here are relatively modest. However, it is important to note that these changes were observed after short treatment times. DR is a disease that develops over years, allowing BM alterations ample time to develop, grow, and influence the behavior of the vascular endothelium. When these changes are extrapolated over time, one can appreciate how even slight changes can accumulate to create larger, clinically relevant problems. In summary, we provide evidence of a number of novel conclusions; included are: inflammation plays a key role in stimulating altered ECM expression by hRMEC and hRP, inflammation-conditioned ECM alters hRMEC-leukocyte interactions, and pericytes contribute significantly to the development of BM thickening and the subsequent altered cell–matrix dynamics.
